# Assessment of genetic diversity of an endangered tooth-carp, *Aphanius farsicus* (Teleostei: Cyprinodontiformes: Cyprinodontidae) using microsatellite markers

**DOI:** 10.22099/mbrc.2017.24404.1246

**Published:** 2017-12

**Authors:** Sareh Yaripour, Hamid Reza Esmaeili, Ali Gholamhosseini, Mohammad Rezaei, Saber Sadeghi

**Affiliations:** 1 Zoology Section, Department of Biology, College of Sciences, Shiraz University, Shiraz, Iran; 2 Fishery Faculty, Gorgan University of Agriculture Science and Natural Resources, Gorgan, Iran

**Keywords:** Maharlu Lake, Genetic differentiation, Microsatellite, Tooth-carp fishes

## Abstract

Genetic structure of an endemic tooth-carp fish, *Aphanius farsicus* from four different water bodies in the Maharlu Lake basin was investigated by applying five microsatellite markers. All of the five examined microsatellite loci showed polymor-phism pattern. A total of four alleles were detected at five microsatellite loci, with an average of 2.8 to 3.5 alleles per locus. Average values of observed and expected heterozygosity were 0.95±0.09 and 0.64±0.02 respectively. None of the tests of linkage disequilibrium were significant between each pair of loci and no deviation from Hardy-Weinberg equilibrium were detected to test for heterozygote deficiency within populations. The Nei's genetic distance values ranged between 0.03 – 0.13. Analysis of pairwise genetic differentiation between each pair of the populations revealed that fixation index (*F*_ST_) values ranged from 0.013 to 0.039 and R_ST _ranged from 0.005 to 0.065. High genetic diversity observed within the populations (99%) and low diversity (1%) among them indicating probably high level of gene flow among the studied populations of Fars tooth-carp at the present time or in the past. Regarding low genetic differentiation among the studied populations and results of population assignment test, two hypotheses are suggested and supporting evidence for each hypothesis are provided.

## INTRODUCTION

Species of the killifish genus *Aphanius* Nardo, 1827 are widely distributed in coastal habitats of the Mediterranean Sea, Red Sea and Persian Gulf, as well as brackish, freshwater and euryhaline inland water bodies in Iran, Pakistan and India [1-2]. To date, 14 *Aphanius* species have been described from Iran, including *Aphanius farsicus *Teimori, Esmaeili & Reichenbacher, 2011 an endemic cyprinodont fish restricted to the Maharlu Lake basin in Fars Province, southwestern Iran [3]. However, this species has received little scientific attention to date. Keivany and Esmaeili (2013) [3] suggested that the species should be in the IUCN’s Red Data Book due to criteria such as restricted distribution, destruction of spawning grounds, environmental pollution and drought. Based on our observation, there are several scattered patches of this fish species in spring systems around the Maharlu lake basin isolated from each other by land or by hyper saline lake. These spring-streams may be connected via the temporary brooks during rainy winter season. This arises the questions whether these population patches are related, or is there any barrier to gene flow among the individuals in the studied locations? For answering such questions, implementation of molecular markers has highly been recommended. 

Among molecular markers, microsatellite methods are widely applied for population genetics due to high levels of variability, the ability to investigate this variation using PCR technology, codominant nature of Mendelian inheritance, their abundance in genomes and small locus size. Also, microsatellites are useful in studies of endangered species because small amounts of tissue are required [4-8]. So far, these markers have been used to determine genetics differentiation between two populations of *A. vladykovi *in Iran [9]. In the present study, we used five previously designed microsatellite markers to estimate the level of genetic diversity and to compare the degree of genetic differentiation among *A. farsicus *populations collected from four water bodies in the Maharlu Lake basin, southwestern Iran. Our results about the genetic structure of this species will be used to define management programs with attendant implications for conservation.

## MATERIALS AND METHODS

**Figure 1:**
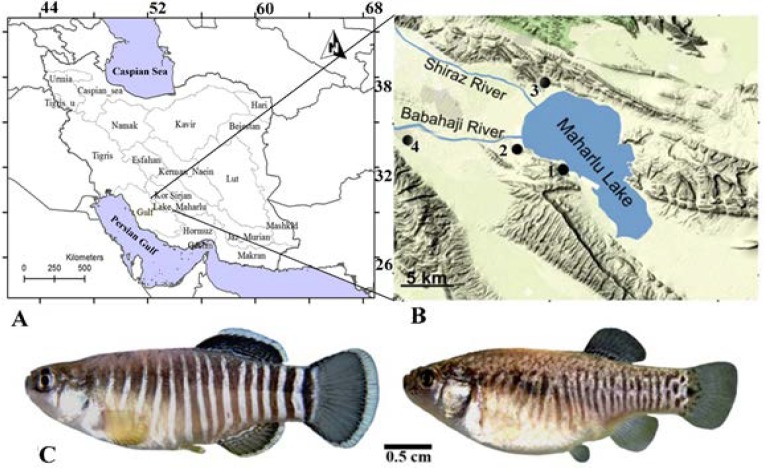
A,B) Sites of the collected populations of *Aphanius farsicus* (1- Dobaneh, 2- Barmeshoor, 3- Barme Babunak, 4- Pirbanoo). C) Male (left) and female (right) of *Aphanius farsicus* from the Barmeshoor spring-stream (modified from Gholami et al., 2015).

**Sample collection and DNA extraction: **To assess genetic variation in *A. farsicus*, a total of 23 samples were collected from spring-streams Dobaneh (29º 25′ 48.1′′ N; 52º 46′ 24.7′′ E, altitude 1453 m), Barmeshoor (29º 27′ 9.51′′ N; 52º 42′ 0⋅051′′ E, altitude 1465 m), Barme Babunak (29º 33′ 58.68′′ N; 52º 44′ 23.2′′E, altitude 1480 m) and Pirbanoo (29º 28′ 12.72′′ N; 52º 30′58. 68′′E) in the Maharlu basin, southern Iran during 2011-2013 (Fig. 1) [10]. As the species is currently found only in the Maharlu Lake basin, southwestern Iran; therefore we took a few number fish due to conservation considerations in this study. All collected specimens were deposited in the collection of the Zoological Museum of Shiraz University, Collection of Biology Department (ZM-CBSU). Total genomic DNA was extracted from fin tissue using standard phenol/ chloroform extraction procedures [11]. The quality of the extracted DNA samples was checked by 1% agarose gel electrophoresis [12]. 

**Polymerase chain reaction and electrophoresis: **PCR amplifications were done using five microsatellite loci that had been previously optimized for *A. fasciatus* (Af7, Af20, Af20b, Af61 and Af18) [13]. The PCRs were performed in 10 µl volumes containing 0.5 U of Taq DNA polymerase (Fermentas), 1.2 µl of 10 X PCR buffer (CinnaGen, Iran), 0.25 mM dNTPs, 2 mm MgCl_2_, 0.5 µM of each primer and about 50 ng template DNA. The amplification consisted of an initial denaturation at 96°C for 3 minutes; followed by 30 cycles of denaturation at 94°C for 30 s, primer specific annealing (Table 1) for 30 s and extension at 72^◦^C for 30 s and a final extension at 72^◦^C for 10 minutes. PCR products were electrophoresed on 8% polyacrylamide gels and followed by silver-staining [14]. Microsatellite allele lengths were estimated using Gel-Pro Analyzer 3.9 software. Each gel contained an allelic ladder (50 bps) to assist in allele scoring.

**Table 1:** Characteristics of five microsatellite loci used in this study

**locus**	**Primer sequences (5-3) **	**Annealing temperature (** ^˚^ **C)**	**Repeat motif**	**GenBank Accossion no.**
Af7	F: GGAAGCACACATTCAAAACC R: TGTGAGGTCAGAAAGGGAGA	54.3	(CA)14	(DQ865156)
Af20	F: GGAAGCTACAAGGGATGAAA R: GCATGCTGGCAATTCCATAT	49.8	(CCAT)7TCCT(CCAT)3	(DQ865160)
Af20b	F: GAGGCTCACTAATCCACTCT R: TCAATTACCAAAGCAGGGCT	58	(CA)11	(DQ865161)
Af61	F: ATTTGTTCTGTCTGGGTCAC R: TGAGTCCTGAGTAAGTAACT	54.7	(GT)14	(DQ865163)
Af18	F: CCAATATACACATCTACACG R: TTGTCTCTTTTCTTCTGCAG	48.1	(CA)14	(DQ865159)

**Microsatellite data analyses: **We assumed each of different sampling location as a population because these locations are isolated from each other by land or by saline lake. Scoring errors and presence of null alleles were checked employing the program MICROCHECKER [15]. The number of alleles per locus (*Na*), effective number of alleles (*Ne*), observed heterozygosity (*Ho*), expected heterozygosity (*He*) and genetic distance (D) were calculated using POPGENE ver. 1.31 [16]. Deviations from Hardy-Weinberg equilibrium within populations (with exact *p* values being estimated using the Markov chain algorithm with 10,000 dememorization steps, 100 batches and 1,000 iterations and to test for heterozygote deficiency), linkage disequilibrium between each pair of loci and significant differences in allele frequencies between populations were tested by online version GENEPOP (http://genepop.curtin.edu.au/). Genetic differentiat-ion among the sampled populations was evaluated using *F*_ST_ and *R*_ST _values based on AMOVA (Analysis of Molecular Variance) with 999 permutations using GenAlEx 6.3 [17]. Population assignment test was conducted with GenAlEx, with leave one out option.

## Results

In this study, all five examined primers for *A. farsicus *were successfully amplified and all showed polymorphic pattern. Based on the MICROCHECKER results, no evidence was seen for large allele dropout or stutter-band scoring at any of the five loci. A total of four alleles were detected for all populations and allele sizes ranged from 104 to 235 bps. Allele frequencies at the all loci in the four populations are shown in Table 2. Pairwise comparisons among populations showed no significant differences in allele frequencies at the 5% level using Fisher's exact test (sample size of populations is equal). Overall, four alleles resulted in five microsatellite loci, that alleles 1, 2, and 3 observed in all of the five microsatellite loci, but allele number 4 observed just in three loci (Af20/ Af20b and Af18) (Table 2). The locus Af20 in Barmeshoor and Pirbanoo, Af20b in Dobaneh and Barmeshoor and Af18 in Barmeshoor presented the highest number of alleles (four alleles), while the locus Af20b in Barme Babunak was the lowest (two alleles) (Table 3). Number of alleles and effective number of alleles as well as expected and observed heterozygosity at all loci in the four populations are presented in Table 3. The average number of alleles found per locus in Dobaneh, Barmeshoor, Barme Babunak and Pirbanoo samples were 3.2, 3.5, 2.8 and 3.2 respectively. 

**Table 2:** Allele frequency in five microsatellite loci used in this study

**Loci/ Allele**	**Af7**	**Af20**	**Af20b**	**Af61**	**Af18**
1	0.19	0.04	0.13	0.10	0.08
2	0.41	0.34	0.52	0.50	0.06
3	0.39	0.45	0.04	0.39	0.47
4	0.00	0.15	0.30	0.00	0.37

The average observed heterozygosities (*Ho*) for Dobaneh, Barmeshoor, Barme Babunak and Pirbanoo regions also were 0.97±0.07, 0.97±0.07, 0.90±0.22 and 0.96±0.08 respectively. Whereas, the average expected heterozygosities (*He*) for these regions were 0.62±0.04, 0.72±0.03, 0.59±0.04 and 0.68±0.03 respectively. The differences among the populations were not statistically significant (P<0.05) in Wilcoxon-Mann-Whitney test, neither for the average number of alleles nor for the average observed and expected heterozyogosity. None of the test of linkage disequili-brium were significant and no deviation from Hardy-Weinberg equilibrium were detected at 95% confidence levels to test for heterozygote deficiency.

Analysis of pairwise genetic differentiation between each pairs of the populations revealed that *F*_ST_ values ranged from 0.013 to 0.039 and R_ST _ranged from 0.005 to 0.065 (Table 4). The *F*_ST_ and R_ST _values from pairwise comparisons were not significant (p<0.05). The *F*_ST_ values indicate the low genetic differentiation among populations that could be explained by high level of gene flow in the past times or connectivity among populations at the present time. Based on results obtained from AMOVA, high genetic diversity observed within the populations (99%) and low diversity (1%) among them. Genetic distance and similarity based on the Nei's index among the populations are summarized in Table 5. The most identity was seen between Pirbanoo and Dobaneh (0.96), then between Pirbanoo and Barmeshoor populations (0.93). The least identity was between Barmeshoor and Barme Babunak populations (0.87).

**Table 3:** Genetic diversity parameters for five microsatellite loci in *Aphanius farsicus.*

**Populations**		**Af7**	**Af20**	**Af20b**	**Af61**	**Af18**
**Pirbanoo**	*N*_a_	3	4	3	3	3
	*N*_e_	2.77	2.94	2.63	2.38	2.38
	*H*_o_	0.80	1.00	1.00	1.00	1.00
	*H*_e_	0.71	0.73	0.68	0.64	0.64
**Barmshoor**	*N*_a_	3	4	4	3	4
	*N*_e_	2.88	3.13	2.88	2.66	3.42
	*H*_o_	0.83	1.00	1.00	1.00	1.00
	*H*_e_	0.71	0.74	0.71	0.68	0.77
**Dobaneh**	*N*_a_	3	3	4	2	3
	*N*_e_	2.66	2.32	2.40	2	2.32
	*H*_o_	1.00	1.00	0.83	1.00	1.00
	*H*_e_	0.68	0.62	0.63	0.54	0.62
**Barme Babunak**	*N*_a_	3	3	2	3	3
	*N*_e_	1.94	2.32	2.00	2.32	2.32
	*H*_o_	0.50	1.00	1.00	1.00	1.00
	*H*_e_	0.53	0.62	0.54	0.62	0.62

**Table 4:** Analysis of genetic differentiation between each pairs of *Aphanius farsicus *populations based on estimates of *F*_ST_ (above diagonal) and *R*_ST_ (below diagonal).

**Samples**	**Pirbanoo**	**Barmshur**	**Dobaneh**	**Barme Babunak**
**Pirbanoo**	-	0.000	0.000	0.013
**Barmshur**	0.000	-	0.014	0.039
**Dobaneh**	0.000	0.008	-	0.034
**Barme Babunak**	0.065	0.055	0.005	-

**Figure 2:**
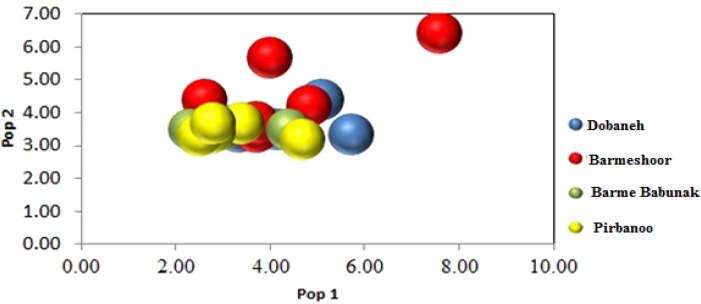
GenAlEx assignment plot showing one clustering of all four sampled populations. Axes are log likelihoods of population assignment.

Population assignment outcomes to 'self' or 'other' population on the basis of negative log likelihood suggested that 65% of individuals were assigned to other populations indicating the close genetic resemblance observed between these sampled locations (Fig. 2).

**Table 5:** Matrix of genetic distance (under diagonal) and genetic identity (above diagonal), (Nei, 1978) between each pairs of *Aphanius farsicus *populations detected at five loci

**Populations**	**Pirbanoo**	**Barmshoor**	**Dobane**	**Barm e Babunak**
**Pirbanoo**	-	0.93	0.96	0.92
**Barmshoor**	0.07	-	0.91	0.87
**Dobaneh**	0.03	0.09	-	0.91
**Barme Babunak**	0.07	0.13	0.08	-

## Discussion

In this study, genetic diversity and relationship among four populations of *A. farsicus* is investigated based on the five microsatellite loci around the Maharlu Lake. The mean number of allele per locus for the four populations was 3.25±0.25, which is lower than the reported N_A_ for freshwater fish (N_A_=9.1±6.1 averaged from 13 species) [18] which probably is caused by the small size of our sampling. The frequency of heterozygosity reflects diversity; therefore, heterozygosity is a useful measure of genetic diversity in a population [19-20]. In this study, the average observed heterozygosity and expected heterozygosity were 0.95±0.09 and 0.64±0.02 respectively, which showed high level of heterozygosity in the four studied populations of *A. farsicus*. In terms of heterozygosity, our results are higher than the average reported for freshwater fish (Ho=0.54±0.25) [7]. Hossaini et al. (2013) [9] reported average observed heterozygosity *in A. vladykovi *(0.96) which is in accordance with the results of this study. Regarding the obtained heterozygosity in this study, high genetic diversity of *A. farsicus* could be a result of natural reproduction, and absence of fishing activities for this species, although some habitat destructions by human and drought in recent years have affected on *A. farsicus* populations. Heterozygote deficiency could result from a subdivision of the local population into isolated and differentiated reproductive units or inbreeding through the mating of related individuals [21]. None of the loci had heterozygote deficiency in the four studied populations suggesting that these populations are not completely isolated from each other. 

Analysis of Molecular Variance (AMOVA) is a scale for determining the level of genetic differentiation and genetic similarity between populations [22]. In the present study, high genetic diversity observed within the populations (99%) and low diversity (1%) among them. Results of Analysis of Molecular Variance also showed low genetics differentiation between two populations of *A. vladykovi* [9]. In population studies, the *F*_ST_ index is an important measure to examine and determine population differentiation [23]. There is low differentiation between each pair of the populations according to Wright (1978) [24] that states *F*_ST_ values from 0 to 0.05, indicate low level of genetic differentiation. Wachirachaikarn et al. (2009) [25] suggested that R_ST_ is more powerful than *F*_ST_ in estimation of population differentiation. Results of this study did not show any significant difference between *F*_ST_ and R_ST_ values. In consistent to our study, Gholami et al. (2015) [10] reported high genetic connectivity among the populations of Dobaneh, Barmeshoor and Barme Babunak, based on *Cytb* mitochondrial gene. 

The genetic distances obtained in this study based on pairwise population comparisons, coupled with the results of ANOVA and population assignment analysis suggested one genetic population cluster. Regarding to the results, two hypotheses are suggested here. The first probability is that annual floods to the lake make a fresh water area in some parts of it, and fishes can be migrated from one spring to another. The lake is drained by incoming fresh water from rainfall and seasonal floods. Therefore, in a short period of time, the waters of northern and western parts of the lake (Fig. 1) probably are partly saline or even fresh. *Aphanius farsicus* can tolerate some degrees of salinity, so during this time, it may migrate from one spring to another. 

The second probability is that currently isolated populations of *A. farsicus* may be relicts of a recently widespread species around the Maharlu Lake and therefore, isolated populations did not have enough time for differentiation and form part of a single genetic population. Study of Gholami et al. (2015) suggested that *A*. *farsicus *has experienced episodes of demographic expansion (*e*.*g*. after a bottleneck probably due to drought in 1967), and provided evidence that present isolated populations of the species might be caused by recently droughts.

The findings of this study on the close genetic resemblance among populations of *A. farsicus* around the Maharlu Lake provides a major issue in the context of habitat management and conservation of this endangered species.
